# On the Diagnosis and Treatment of Morbid Impulse

**Published:** 1863-04

**Authors:** W. Carmichael McIntosh

**Affiliations:** Murray's Royal Asylum, Perth.


### Art. X.—ON THE DIAGNOSIS AND TREATMENT OF MORBID IMPULSE.

By W. Carmichael McIntosh, M.D., Murray's Royal Asylum, Perth.

(*Continued from No. IX. p.* 123.)

The diagnosis of morbid impulse, where the intellect of the patient is little affected, involves at once questions of serious moment and considerable perplexity. On the medical decision oftentimes hangs the life of a person irresponsible for the act he has committed, or the escape of a degraded criminal justly meriting retributive justice. Again, we may shield from public contumely females—even ladies of station—who instead of committing a breach of the laws, are solely impelled by a morbid and irresistible impulse. A difficulty has been started by some as to whether or not all crimes are not due to some morbid state of the instinctive or other faculties; and this, amongst other things, has led some writers to deny the existence of impulsive mania. In regard to the medico-legal bearings of the subject, scarcely a year passes without the importance of a skilled and scientific knowledge and experience of this form of insanity being fully appreciated and recognised. The case of Mrs. Vyse, so recently tried, is a fair example. Without apparent motive she poisoned her children, and then attempted suicide. The decision of the court was in keeping with the principles of psychology, for she was sent to St. Luke's. It not unfrequently happens, however, that the legal element in the criminal courts, and the people generally, are averse to medical jurisdiction in the matter, imagining that either sinister purposes or ignorance are at the root of the medical testimony, calculated to frustrate the demands of a violated law and the clamour of outraged justice. So absurd are the opinions of lawyers upon this point, that they maintain that they are capable of judging of a man's responsibility or irresponsibility by the gauge of "knowing right and wrong," and affect to disregard medical testimony to the contrary. They place their opinion and powers of diagnosis before those whose lives have been devoted to the study of mental and physical diseases, and whose entire pursuits alone fit them to form or conduce to a right judgment of the question. "Medicine maintains that a theoretical and practical study of mental diseases and defects is necessary to the proper understanding and detection of mental disease or defect; law denies this, and says that it is a fact to be determined by any dozen of ordinary men in consultation on the case. Medicine says a man may be insane and irresponsible, and yet know right and wrong; law says a knowledge of right and wrong is the test of both soundness of mind and responsibility to the law. Medicine says, restrain and cure the insane and imbecile offender against the law; law says, hang, imprison, whip, hunger him, and treats medical art with contempt."* It is well known, however, that when a court sits to deliberate on a case of suicide, the dogma of "right and wrong" is wonderfully pliable, and verdicts of "temporary insanity" are the rule.

The friends of the patient too frequently delay bringing the case under medical notice, shrinking from the stigma so vulgarly associated with all forms of what is called "insanity," and hence it so often happens that some act has been committed which complicates and perhaps renders the case a subject of medico-legal investigation. Diagnosing under such circumstances may be likened to the bringing of a patient to us for the first time, who has vomicæ, cavernous respiration, and amphoric resonance, the result of insidious yet noticeable disease. All psychologists are so fully alive to the faintest indications of such a tendency to mental disease, and to the circumstances which engender it, that they lose no opportunity of inculcating the importance of attending to its early manifestation. And the necessity of, and incentives to, such a course are manifestly evident; to save a family from disgrace, sorrow, and even destruction, and to protect both the individual and the public from injury, are laurels worthy of a struggle. In this, as in physical disease, the great point is the early recognition of the tendency. The terrible results which sometimes ensue on the neglect of premonitory symptoms are shown in the following cases.

Some years ago a tragedy occurred in the north of Scotland, which filled the newspapers of the day with bloody details. One evening, an esteemed and respectable man, with a wife and large family, retired to rest, to unobservant eyes appearing just as he ever did; but suddenly, during the night, evincing the most intense homicidal impulse, he savagely slaughtered his wife and his children, with the exception of one, who mercifully escaped

* Prof. Laycock: *Edin. Med. Journal,* June, 1862, Introductory Address.

from the impulsive maniac, and then finished his work of destruction by suicide. Whether this was one of those cases having connexion with sleep and dreaming, I am not aware; but hereditary taint was present, since a near relative appeared in the Royal Infirmary under Professor Laycock, in a moody and melancholic condition. Some premonitory symptoms were doubtless noticed by the man's friends and relatives previous to the committal of these fearful acts, a careful attention to which might have averted this lamentable event. A somewhat similar case occurred recently, in which a sergeant of artillery murdered with desperate accuracy his wife and numerous family, and unsuccessfully attempted his own life. In this instance there were marked premonitory symptoms, and it was astonishing that no means were taken to guard against such a terrible calamity. Such cases as these—and they are numerous—speak for themselves. The advantages, on the other hand, of strict and scientific appreciation of symptoms is seen in the case of the gentleman with the ascarides, narrated at p. 116; of the patient and the vegetables, at p. 115, and many others. Numerous acts of impulsive insanity might be prevented, were the physician always to keep in view the possibility of the symptoms, which, perhaps, he treats so lightly, being the precursors or actual manifestations of brain disease, in which premonitory stages these impulses are frequent. The premonitory symptoms, it is true, are sometimes not very well marked even in striking cases of impulse, and are apparently wanting in some. "So cleverly and successfully is the mark of sanity and mental health sometimes worn, so effectually is all suspicion disarmed, that mental disorder of a dangerous character has been known for years to be stealthily advancing without exciting the slightest notion of its presence, until some sad and terrible catastrophe (homicide or suicide) has painfully awakened attention to its existence."* Nor is it in the outer world alone that the psychologist has to be a close observer of such tendencies. Cases of undoubted insanity under his charge in a lunatic hospital are equally objects of solicitude, for it is not a very uncommon thing for homicidal and suicidal impulse to be developed suddenly in connexion with other forms of brain disease, and unless attendants are duly warned in such cases, serious results might ensue. For instance, a female patient, aged sixty-six, of highly arthritic diathesis and more or less hemiplegic, was admitted into an asylum suffering from senile dementia, and with no definite strictures in regard to suicide, though further information showed that she had attempted it.

* Dr. Winslow: *Obscure Diseases*, &c., p. 142.

For nine months she conducted herself very quietly, exciting not the slightest suspicion. Suicidal symptoms were not upon any one occasion manifested or observed; so much, indeed, did she win the confidence of the physician, that he had taken steps to have her removed, and if they had been properly attended to, she would have been free some weeks before the fatal occurrence. A visit from her son, who, contrary to her expectations, gave her no hopes of going home with him, seems to have excited the impulse afresh, for on the following morning, though in her usual health and spirits, she hung herself from the water-closet door by means of a very ingenious contrivance. In consideration of her very frail condition, she was permitted the use of an umbrella, as a means of support to her in walking; to the handle of this she tied a common cotton neckerchief very securely, although quite unable to dress herself, and having done so, passed the free end of the neckerchief through the inspection-aperture of the said door from without, the umbrella forming a very efficient check to the suspensor. She then adjusted the free end of the neckerchief around her own neck without undoing any article of dress, her cap and bonnet remaining on her head, and leant the weight of her body on her neck by pushing her feet away from the door. She was quite dead before she was discovered, with her feet of course resting on the ground.*

In cases where the physician is voluntarily consulted by patients who have diseased viscera, and at the same time experience what they cannot account for: viz., an almost uncontrollable impulse to commit some act—it may be criminal, such as that of homicide or suicide—or, if there is nothing more than a general feeling of distress and alteration of previous habits, the greatest attention must be paid to such symptoms,

---

* In the post-mortem examination, the cranium was found of great thickness, with its diploë and tables not well defined, and readily broken with the bone-forceps; dura mater adherent; slight effusion of blood over the posterior part of both hemispheres of the cerebrum; brain substance softened throughout, especially around the lateral ventricles, where it was almost pulpy; the lining membrane of the lateral ventricles covered with a large amount of lymph exudation, thickest around the venæ c. striat. There was a circumscribed greyish, softened portion in the right cone, close to the pons; cerebellum also softened, the left hemisphere having its dentritic structure rendered indistinct; arteries throughout atheromatous, and just where the ophthalmic sprang from the internal carotid on the left side there was a small true aneurism about the size of a pea, pressing slightly on the optic nerve; total weight of encephalon, 47 ozs., of which the cerebell., medull., and pons weighed 6 ozs.; blood throughout entirely fluid. The microscope showed disintegrated nerve substance, granules, and atheromatous vessels at the softened portion of the brain; upper part of spinal cord much softened.

It is an interesting fact in regard to this case that a fellow-patient entered and used the water-closet while the suicide was suspended, without giving herself the slightest concern, and, as she afterwards explained, that she thought it best to say nothing about it!

especially where brain disease is probable. Instances might be cited, on the one hand, where valuable lives have been saved by careful and accurate diagnosis; and, on the other, by neglect or misconstruction of the symptoms, most serious results have ensued. It is stated, curiously enough, that we may find the tongue clean, the pulse regular and tranquil, the head cool, and the secretions normal; yet the hands of such a patient may be imbrued in the blood of a father, a mother, a wife, or a child.

The incipient stage or period of incubation is certainly, in relation to diagnosis, the most important one, both as regards the cure of the patient and the welfare of his friends. We see an individual, perhaps with a slight hereditary taint, from his earliest years displaying the most lax morality on one or more points, but who is by no means different from his neighbours in other matters. If in such an one we observe extravagances, or any of the minor impulses, or a tendency thereto, perhaps unnoticed by his nearest relatives, with a certain peculiar solicitude or moroseness—it may be only temporarily—we would take precautions to avert the onset of morbid impulses, which are surely imminent. Again, it is often observed that the habits, tastes, and passions change; the calm, steady man of business becomes extravagant and reckless, indulging in wild speculations, which are generally unfortunate; and the consequent reverses, instead of being the cause of his disease, are its result. If in a child of drunken or insane parentage a craving for alcoholic stimuli is noticed, it might be inferred that, as years roll on, so the habit will become impulsive and swell into gigantic proportions—as dipsomania—or else hurry the unfortunate individual into more criminal acts. Where there is a strong hereditary taint the morbid impulses are seldom single, and we have excessive sensualism, dipsomania, &c.; in short, the moral faculties and instincts in general depraved and perverted. Well has it been said, "that the faintest symptom of disease in the viscera or other organs at once attracts attention; but how often do we neglect alterations of temper, depression of spirits—amounting sometimes to melancholia, headache, severe giddiness, inaptitude for business, loss of memory, confusion of mind, defective power of mental concentration, the feeling of brain lassitude and fatigue, excessive ennui, a longing for death, want of interest in pursuits that formerly were a source of gratification, restlessness by day and sleeplessness by night—all obvious indications of an unhealthy state of the functions of the brain and nervous system; rarely, if ever, do these attract attention until the disordered mind rushes into homicide or suicide." Almost every one of the criminal cases of morbid impulse come under this category, leading us to suppose that if the diagnosis had received more attention, a better fate might have resulted to the actors. To attract notice to the incubation of those forms of insanity, an able pen thus writes : " A person loses confidence in the talents and prospects which he had previously regarded with satisfaction, and he gives his thoughts up to ruin and desolation. When the individual has the fortitude to control, or the cunning to conceal the expression of the full extent of his sufferings, it is called lowness of spirits. It ought to be regarded as insanity, and not dreaded as its forerunner ; for it is at this stage that suicide has resulted. . . . . However imperceptible, there is always a premonitory stage, such as bodily disorder, mere unsteadiness of purpose, or irritability of temper, or craving for excitement ; but it is as much part and harbinger of the disease as the cold is of the hot stage of intermittent fever."

In actually examining the patient suspected to be labouring under impulsive insanity, similar conduct and bearing is to be observed as in ordinary cases of insanity ; and even greater caution is necessary where the persons are acute and cunning, and wish to conceal their defect. By careful inquiries or conversations we should endeavour to draw the individual to exercise the particular instinct, passion, or emotion of which we fear there is exaltation, perversion, or degradation ; but this is often a difficult task, of which the following case is an illustration. A military officer, who during his life had been remarkable for vigour and superior intellectual endowments, manifested a strong desire for the death of his servant and others for, as he imagined, poisoning his food. His friends, amongst whom was a medical man, considering their lives perilled, introduced several distinguished medical practitioners into his house, with a view of noting his insane bearing and conduct, and certifying accordingly. Although ignorant of the exact nature of their visit, the patient mingled with the medical men for hours without manifesting any symptom of violence or insanity ; in fact, all were astonished at the amount of information and correct judgment he displayed during ordinary conversation, or in the recital of his adventures. Wearied with the fruitless effort, the party sat down to dinner, at which there happened to be a particular kind of dark soup. One of the medical men, knowing the exciting cause of the patient's outbursts, adroitly hinted that the soup was not good, and in fact that he felt ill. Immediately the officer, thrown off his guard, broke into a violent paroxysm of rage, threatening the life of his servant. Suspicion, however, flashed across his mind respecting the company he was in, he checked himself suddenly, and conscious of having overstepped the boundary of caution, burned to avenge himself on the party who had been instrumental in betraying him.

A complete history of the patient, if it can be accurately obtained, will materially assist us in forming our diagnosis; sometimes the friends of the patient are anything but correct in such particulars as may be of most importance. The family history will also often throw light on an otherwise obscure case, as was recently illustrated in the London tragedy of Mrs. Vyse. The disposition and temper, where not congenitally defective, often shows marked changes in incipient impulse. We must carefully consider all the circumstances mentioned under the head of causes, and with caution we seldom fail to arrive at a definite and trustworthy diagnosis. It has been frequently observed that a person having a terrible impulse hanging about him is sad and melancholy; the due appreciation of this brooding may preserve the patient and the public from injury. Instead of a melancholy condition, there may be exuberant gaiety, the result of having secured an effectual means of accomplishing the purpose. In the case of one who has already carried out an impulse, we must regard the place and circumstances, motives, and previous history, family and individual, assisted in our investigations by disinterested friends or relatives. We must also inquire into the amount of education received by the patient, and the manifestation or absence of intellectual, moral, or merely animal vigour; and if his thoughts and powers of judgment remain as they formerly were. In asylum practice, the value of a correct diagnosis is considerable in cases which, while generally quiet and inoffensive, have a tendency to sudden impulses. The responsibility of the physician who neglects the premonitory symptoms of such outbursts may be great, where otherwise a timely caution would have averted mischief. The constant warnings necessary, even in overt tendencies of this kind, show how difficult and full of anxiety such cases are. A scientific appreciation of the premonitory symptoms in doubtful cases is the only safeguard. Secret masturbators and females who masturbate unobserved, would often reach an alarming degree of exhaustion if the vice were not otherwise detected than by the sense of sight; and so with the other varieties of morbid impulse.

The physical causes render it imperative that a careful examination be made of the body of the patient, such as for scars of wounds about the head, and the general configuration of the same; the state of the pupils, gastro-intestinal system, viscera of the chest, the liver, urine, generative system, and the ascertaining whether self-abuse is practised. The sensibility of the skin, presence of skin diseases, and the muscular power of the body generally, merit attention.

In regard to the *medico-legal* diagnosis, the principles enunciated as to the causation and nature of the disease are to be borne in mind, as well as the incentives which would have led a sane man into like criminalities. In no department of medical science has there been so much trouble experienced by the physician as in the diagnosis of impulsive insanity before a court of law. Even to this day, the illiberal and ignorant practice in some high quarters of slighting the testimony of skilled medical witnesses cannot be too strongly condemned. When any important and intricate case of poisoning happens, do not the authorities seek the advice and help of skilled and experienced analysts ? And there is no reason why it should be so different in cases where much more extensive experience and culture is needed. This "antagonism" of law and medicine as to insanity, has been ably set forth by Professor Laycock, in his introductory lecture for last session (1862). Speaking of the "knowledge of right and wrong," as a criterion in law courts, he observes:—"Daily experience rightly read, as well as medical science and experience, abundantly shows that a man or woman may be imbecile morally from cerebral disorder and disease, and yet may have good intellectual, nay, high logical powers. There are many who, being thus diseased mentally, drink to drunkenness, fornicate, lie, steal; are obscene, homicidal, cruel, malicious—in spite of a knowledge of right and wrong, and with the reasoning powers little, if at all, affected; and whatever the law may decide to the contrary, the inexorable logic of facts will hold its own. It is in vain alarmists and opponents of these facts tell you, that there are more drunkards than would fill existing asylums thrice over;* in vain they say, if you treat every imbecile knave as irresponsible, you must convert all gaols and prisons into asylums; in vain they express their alarms that if these doctrines be admitted as true, the foundation of the social fabric will be shaken; the truth is not less the truth, and I take leave to say, that until it is carefully inquired into by our legislators, and made available to the reformation or proper restraint, rather than the punishment of imbecile criminals, the same scandalous routine will be followed with the criminal population as hitherto, and which is contrary to even the simplest principle of Christian morals. The question is one in which medical science, ethics, and common sense are in perfect accord. It may be laid down as a first principle, that the capacity of an

* Or as a dipsomaniac whom I knew, when advocating his release, invariably had it (probably copying from the newspapers), "would cover acres of ground."

individual to be influenced by the motives which influence the average of mankind in health and soundness, is the measure of his moral responsibility to society and of society to him. He may be a mere child in moral development and in judgment, and when this is proved in the case of an idiot or congenital imbecile, the plea of irresponsibility to society is admitted, and society becomes responsible for him and to him, and keeps him out of harm's way. In like manner, the cases of notional, impulsive, and vicious imbeciles might be treated; the capability of self-control being the practical question to be decided by a jury, and not the amount of knowledge. Thus, for example, in the case of an alleged vicious lunatic, the question to be raised is not whether he is insane or not, but whether he is capable of controlling his impulses to vice or not."

In considering any particular case, the fact that the crime frequently occurs, irrespective of morbid impulse or insanity, should always be borne in mind. A man may be an inveterate drunkard and yet not mad; and a vagrant may set fire to a public building or a farm-yard without being a lunatic. A lady, even in genteel circumstances, may pilfer, urged by another cause than inevitable impulse;* murder does not always bear the extenuation of insane homicide. The state of the patient must be generally investigated by the physician, and his interviews frequent. A description of the act from the person himself, and his reasons, together with all those niceties of causation and diagnosis which have been sketched in previous pages, will generally enable the alienist to judge correctly. The crime being the result of impulse, the mind may manifest no derangement after its commission; and further, a person may commit a murder under the influence of insane impulse and frenzy, and yet become quite sane before the prison-bolts have severed him from the outer world and freedom. Again, a sane person, under the horror of remorse of such an accusation and crime, has become insane. In diagnosing criminal cases, the experienced alienist will observe peculiarities about the real, which distinguish it from the simulated affection. The momentary gratification of the desire does not generally extinguish the existence of it; but not always, as seen in those cases from easily remedied physical excitants. In kleptomania, other peculiarities besides those of theft may generally be noticed, and the patient steals as readily from her own house as from tradesmen's shops. In the homicidal impulse, "there is the intense desire to kill, but at the same time a sympathy for, a wish to save, and an effort to warn the intended victim. There is this desire without any such

---

* See *Journal of Mental Science*, July, 1862, p. 263.

sympathy, but attended with a violent internal struggle to resist temptation. And again, there may be the desire, unmitigated either by sympathy or struggle, burning for gratification, pausing it may be from fear, but ever watching for opportunity."* Further, it is known that impulses of an irresistible kind occur in the state between waking and sleeping, and also in somnambulism; the presence of either of which conditions ought certainly to render the person irresponsible. Great discernment, however, is necessary, for cases of assimulation are not wanting to prove that lawyers, alienists, and judges have alike been duped by such impostors.†

In all cases of impulsive insanity the perverted instinct has ruled supreme, to the exclusion of the higher faculties of reason and judgment, at least for the moment; and no more could such an one be held responsible for the impulsive act than the veriest imbecile or idiot. The great point in dispute is, whether such an one had control over his actions at the time of committal or not. Experience and common sense, and the weight of the highest medical authorities, all concur in stating that medical testimony can alone be relied on in such cases. In fact, a medical jury, rather than the ordinary one, would be more suitable for deciding the responsibility or irresponsibility —sanity or insanity—of any individual. In regard to the present difficulties encountered by medical witnesses in such cases, it has been well said "that no extent of scurrilous abuse which may be levelled against him should influence the expert when called upon to give evidence in cases of alleged criminal insanity, even to the weight of a hair, in the steady, fearless, and unflinching discharge of one of the most important, sacred, and solemn functions that can be delegated to a medical jurist."‡

*Treatment.*—In treating cases of morbid impulse, or a tendency thereto, attention to the causation holds the first place; neglecting this, the alienist may be likened to a surgeon who treats erysipelatous inflammation of the fore-arm very laudably by fomentations and iron, but who ignores the presence of an irritating spiculum of wood or other foreign body lodged in the substance of the palm, dorsum, or finger. Again, only to treat the patient subsequently to the impulsive act, resembles too often the treating pyæmia after it has set in. Wherever the physician is aware that such predisposition to disease of the mental organism exists, every precaution is most urgently

* Dr. W. A. Browne.

† See case in *Psychological Journal*, which occurred in London some years ago.

‡ Winslow on *Obscure Diseases of the Brain*, p. 184.

demanded. And since these tendencies are by no means uncommon, we ought, by a careful and judicious training of our youth, to endeavour to lessen the number, or at least render such when they occur more amenable to treatment. There are times when no man can foresee the occurrence of sudden reverses, disappointments, and grief; but it is in the power of nearly all to have a mind so trained by an ordinary but proper education as to withstand even such severe shocks. The attempt is at least praiseworthy, and is now pretty well known, if not sanctioned by general adoption.

First, then, let us examine into the best means for preventing or modifying these perverted instincts or impulses. It has been stated that childhood has appetites and instincts strongly developed, while reason is feeble and passion unregulated. A proper system of education (strictly so-called) has a tendency to substitute reason for instinct—to develope the former, to hold the latter in check. "If this be neglected, man will grow up a child in all but its innocence and its inability to do evil; his appetites, impulses, and passions are strengthened by indulgence and lack of any restraining influence, his reason and judgment are null from disuse." A parroting system of education, often so common in schools, and not altogether absent in universities, is anything but a proper culture of the mind, as its subsequent results generally attest. A true education should draw out and cultivate the mental and moral powers, and subdue and discipline the appetites and passions. "Each and every faculty should have its due energy and position, each be predominant or subordinate according to its office for the time being, and all act in concert for the good of the whole. A well-balanced mind should be aimed at—such a mind whose judgment is sound and reliable in all common affairs of life. We know that in the mental and moral constitution every neglect of study or discipline, every error or sin, increases the danger and the chance of repetition of the same fault or mistake, and diminishes the securities against their influence."

The education of one part of our community—viz., the female—is often glaringly defective, especially in the higher classes. Instead of so much German and French, Italian and Spanish, painting and music, and even Greek and Latin, besides a multitude of the more ordinary accomplishments, the female should enjoy plenty of exercise in the open air, should not be advised into constrained positions (like an attitudinizer) within doors, and should be cautioned against too much light, fascinating, and exciting novel reading; or, rather, taught by more useful and common-sense studies the emptiness of such a course. By these and other laudable means, some cases at least of suicidal impulse and erotomania, together with much of the hysteria of the present day, would most certainly be avoided, and a more healthy organization transmitted to her offspring. For it has been truly said that Britain owes much of her greatness to the superiority of her women. However uncharitable it may seem, there is no doubt that many of the peculiarities of comparatively young unmarried females, especially where there is a tendency to the imitation of the masculine, are due more or less to a perverted natural instinct, in close relation to the condition of the generative organs. There can be no better proof of this than that in married females of the same age (of the two conditions, the one that is most fitted to develope the natural instincts, emotions, and passions of women) we seldom or never encounter such chimerical notions.

No false ideas of life should lurk in a well-trained mind. One with ideal hopes of lofty ambition, for the attainment of which he has neither the perseverance nor talent, cannot but meet with disappointment, and, it may be, that a series of reverses result, which sink him into hopeless confusion or blind impulse. This, then, might have been avoided by a careful and judicious education, which gives us sober and common-sense ideas of things as they are, and annuls visionary aberrations. Indulgence, it is well known, always strengthens the vice; and how lamentably do we witness this in the votaries to the distorted shrines of Bacchus, opium, &c. The appetites should be indulged and the propensities allowed to act only at such times, and at such periods, and so far as the health of the system requires. By a calm survey of man's position in this world, and the duties required of him, we can form a just notion of his conduct, and the necessity for the government of reason. "Such habits should be acquired as are consistent with just sentiments; to withdraw the nourishment from the very root of passion rather than for ever be troubled with the pruning of its shoots."* These instincts, &c. should be kept in due control, thus increasing the health and happiness, instead of allowing them unbridled to wreak the ruin of the possessor. Our temperaments are widely different, and every one of us has his weak points; it behoves therefore to guard well those salient angles of our moral fortress, lest unawares they fall, and land us in positions of doubt and intricacy. To succeed in so controlling our natural instincts is a triumph worthy of much effort; for "virtue brings her own dowry." There is an inexpressible thrill of satisfaction which shoots through every nerve of one who is conscious of having performed his

* Dr. W. A. F. Browne, *Reports*, &c.

duty, that all the pleasures and allurements of the "world" never can approach, far less equal.

In addition to the foregoing mental training, the corporeal health should by no means be neglected, and the due performance of every function should be attended to, more especially in those predisposed to nervine disease. The want of attention to regular open-air exercise is as much a cause of mental disturbance as of physical ill. Even where the impulse has occurred, this is one of the most important remedial agents. A life of idleness is favourable to the production of diseased impulses, while useful mental and corporeal activity is not.

However indistinct the traces of the tendency to morbid impulse may be to ordinary eyes, the knowledge and experience of the physician should be sufficient guarantees for committing the treatment solely into his hands. As often happens, the tendency to impulse is but the premonitory stage to more serious mental wreck, and therefore the diagnosis and treatment should be both prompt and effective. Whenever it is understood that a person is really defective in his mental organization, so as to render him incapable of distinguishing between right and wrong, and reasoning correctly upon the consequences of his actions, law takes upon itself the protection alike of society and the defective being himself, for it is well known that motiveless and impulsive acts and outrages are common. But in the former case, where the traces to the unscientific or unpractised eye are faint, not only does medical advice meet with serious opposition, but the physician may be subjected to serious lawsuits for having upon his soul and conscience certified that the individual in question requires as much protection for the time being as the hapless idiot and imbecile.

When the act of impulsive insanity has been committed, and the morbid tendency has declared itself alike the offspring and action of a diseased brain, the first duty in most cases is an examination into the physical condition of the patient. It would be altogether unnecessary to commit one to an asylum whose whole disease lay in a bunch of ascarides in the rectum, or some such slight physical disorder. If there are suppressed hæmorrhoids, we try leeches to the anus. All disorders of menstruation, whether of debility or plethora, require attention. In obstructed menstruation, the compound decoction of aloes and the compound galbanum pill are useful. Where the female is plethoric, the compound jalap powder will give relief. In uterine irritation, from whatever cause, hops, camphor, opium, and hyoscyamus may each be of avail. The use of galvanism across the pelvis, through the ovaries, will often succeed in suspended menstruation, when other means have failed. In nymphomania, leeches to the vulva are stated to be beneficial. In those cases in which the morbid impulse is connected with illusions or delusions of hearing and vision, we may apply leeches to the ears or superciliary ridges. The application of counter-irritation, in the shape of blisters, kept open by the ung. sabinæ, may be serviceable. Evidence should be sought lest any of the diseases mentioned under physical causes should exist, and each to be treated accordingly. Exercise of sufficient amount, alternating with appropriate amusement, should be insisted on in those of sedentary habits; for even in well-regulated minds, monotony and confinement are not free from danger, and a change is both agreeable and necessary.

In many of the temporary, and in all the confirmed cases of impulse, the best place for treatment is assuredly one of our public asylums, presided over by medical men of ability and experience. The quietude which the impulsive forms enjoy, by withdrawal from their ordinary routine of business and from their family associations, and the subjection to regular life and careful regimen, is favourable to restoration. In no other position can we so well regulate the quantity and quality of food, and the amount of mental and physical exercise for the patient. We can also best adapt the kind and quality of the patient's reading, for it is often necessary to limit the use of religious books, &c.; the amount of sleep he enjoys at night, or if he is disturbed by dreams, or is noisy and restless. In addition, asylum conveniences afford more readily the cold shower-bath, the warm bath, and seclusion from excitement of all kinds, so necessary to the welfare of the patient. By strict attention to dietetic rules we may be agreeably surprised to find that the impulse has vanished with the patient's debility, and therefore is truly caused by mal-assimilation and poverty of blood. There are few cases, where the patient is weak, that will not be benefited by a good regimen—a result quite in consonance with that which has been previously stated in regard to hunger. Wine, and even stronger stimuli, are stated to benefit such cases, though both sometimes signally fail. Asylum treatment in those having a tendency to dipsomania, and some other perverted appetites and instincts, transforms in a few days a complete wreck of humanity into a rational and it may be a talented individual, though it often betrays other moral lesions of grave import, which, in the ordinary course of events, have been overlooked by the extreme prominence of one leading impulse. In cases of strong suicidal or homicidal impulse, even the best regulated asylums are frequently insufficient to protect the individual or the community, for deadly assault and suicide occasionally occur in spite of precautions, and with the most unlooked-for weapons. Such are always, and justly, sources of much anxiety to asylum authorities. All stimuli calculated to disturb or irritate the impulsive brain should be removed. It has been proposed—I do not know with what result—that in suicidal impulse, the perverted instinct of love of life should be restored by any means which might tend to excite it. If the patient wishes to commit suicide with a razor, it may be checked by suggesting arsenic, with a due explanation of the terrible anguish consequent on such a death. The instinct is aroused from its dormant condition. Such of course would be suitable only for the milder instances. Half successful cases of suicide are thus sometimes curative. The well-known case of the man who, while proceeding to throw himself into the Thames, and who was attacked by an armed footpad, &c., is a good illustration. It is not uncommon to observe in lunatic patients a decided change, for the time being, under typhus and typhoid fevers. I knew a female with a homicidal impulse, often the cause of serious assaults on her companions or the attendants, who, during a smart attack of typhoid fever, became quite gentle and docile, kind and affectionate, and when convalescent, talked much and kindly of her relatives, wondering why she had been so wild and troublesome. In a week or two, however, after convalescence, she again became sullen and incoherent, and resumed all her former obscenity and tiger violence, springing suddenly and treacherously on her victims. I am not aware of any cases where complete recovery followed such a physical disorder, but it is not at all unlikely. If insanity is due to a turgescence of the cerebral vessels, then it may be asked—How is it that in disease of the above nature, where the cerebral vessels are doubly gorged (the female being stout and plethoric), the mental condition is so much benefited? Is the cerebral "lesion" which we hear so much of, patched up for the time being by an increase of poisoned blood; or is the circulating fluid so nicely adjusted under such circumstances to the altered brain texture, that healthy mental manifestations result? When a healthy attendant happens to take a similar fever, his bearing under the disease is not the same; there is a greater tendency to delirium and insane acts.

An impulsive mind is never so safe as when it is fully occupied; therefore intellectual sympathies, in the shape of industrious employments, &c., have a very happy influence on a person in this state. We are familiar with this in a modified form in every-day life. Wild, daring, and mischievous boys often make the most distinguished men in after life, if their intellectual powers are good; and for this reason, that they now lend to a laudable pursuit or profession those profuse spirits and dashing impetuosity which had run to waste previously. A course of study, in some cases, may help to train the errant mind into better balance, while in others, little benefit, and even harm, may result. All the ordinary treatment, such as drawing and painting, music, translating foreign works, turning, garden work, &c., in all cases the mental exercise alternating with plenty of out-door life, whether at game or walking parties. Engaging the mind of the patient in the study of natural history or botany is a practice that cannot either be too highly commended or extensively carried out.

> "With tender ministrations thou, O Nature,
> Healest thy wandering and distracted child,
> Thou pourest on him thy soft influences,
> Thy sunny hues, fair forms, and breathing sweets," &c.
>
> COLERIDGE.

In regard to suicidal and homicidal impulses in asylums, too much care cannot be taken. It will be found that, when these impulses are at their worst, the patient is sleepless and restless at night, and not unfrequently opiates of the ordinary kind are refused or become inert. In such, the subcutaneous injection of morphia or other narcotics by means of Wood's syringe, is of much service both for improving the health of the patient by sound sleep, and abating the impulse. The homicidal always sleep in a single room, for they are not safe in a dormitory, either with or without an attendant, for the latter is frequently assaulted when asleep, with intent to strangle. The suicidal, again, are never allowed to sleep alone, for even with the ordinary bedclothes, and with no higher point of suspension than the bed, fatal mischief may ensue, even should other means be cut off by the vigilance and penetration of attendants. Such will secrete pins, nails, and stones, put strings in their mouths, armpits, and under the bedding, choke themselves with their own hands, or by thrusting the bedclothes into their mouths and over their faces. The only safeguard in many cases is supervision by a special attendant, whose sole duty is to attend to the alien.

